# Correlating the Preoperative Modified Japanese Orthopaedic Association Score With Baseline Motor Evoked Potential to Justify Neuromonitoring in Degenerative Cervical Myelopathy Surgery: A Pilot Study

**DOI:** 10.7759/cureus.103477

**Published:** 2026-02-12

**Authors:** Ahmad Ikhwan Mohd Sharfuddin, Zairul Bahrin

**Affiliations:** 1 Orthopaedics and Traumatology, Hospital Pulau Pinang, George Town, MYS; 2 Orthopaedics and Traumatology, Hospital Kuala Lumpur, Kuala Lumpur, MYS

**Keywords:** cervical myelopathy, degenerative cervical myelopathy, intraoperative neuromonitoring (ionm), modified japanese orthopaedic association (m-joa), motor evoked potential monitoring

## Abstract

Introduction

The ability to predict the measurable baseline motor evoked potential (MEP) in degenerative cervical myelopathy (DCM) condition using a preoperative clinical score such as the modified Japanese Orthopaedic Association (mJOA) score may allow savings in healthcare costs, as intraoperative neuromonitoring contributes significantly to the rise of healthcare costs.

Materials and methods

This is a retrospective case cohort of 22 patients looking at the ability of the preoperative mJOA score to predict baseline MEP in DCM patients undergoing surgery in three regional spine centers over one year, from July 2023 to July 2024.

Results

MEP was indeterminate in 36% of patients. Using univariate threshold analysis and ROC curves, a classification threshold of 71% was chosen, where the mJOA score was 13. Binary logistic regression analysis showed an odds ratio of 1.22 (p=0.22). Every point of increase in the mJOA score leads to 21.76% increase in the likelihood of MEP being present at baseline.

Conclusion

An mJOA score of 13 gives a 71% probability of MEP being present. Increasing the value of the mJOA score increases the likelihood of MEP being present in the lower limb but fails to reach statistical significance. We suggest a greater number of patients while including other preoperative neurologic markers to predict the presence of baseline MEP.

## Introduction

Intraoperative neurophysiological monitoring (IONM), particularly motor evoked potentials (MEPs), is an important modality in modern spine surgery, intended to prevent iatrogenic injury [1]. With healthcare costs soaring, the justification for IONM becomes increasingly nuanced [2]. Its utility hinges on a fundamental prerequisite: a measurable baseline signal. In surgeries for degenerative cervical myelopathy (DCM), where the spinal cord is already compromised, this prerequisite is often unmet [3]. Absent or indeterminate baseline MEPs render IONM ineffective for its primary purpose of injury detection, therefore raising critical questions about its value in a significant subset of patients [3].

This study addresses this dilemma by investigating the modified Japanese Orthopaedic Association (mJOA) score as a clinical marker to predict the usefulness of MEP preoperatively by correlating it with the present baseline signal. The mJOA score, a standard measure of DCM severity, can serve as this crucial guide, enabling surgeons to identify patients for whom IONM offers genuine utility and ensuring both clinically effective and economically responsible outcomes. We hypothesize that a higher preoperative mJOA score correlates with a higher probability of measurable baseline MEP, which could help stratify patients who would benefit from IONM.

## Materials and methods

This was a retrospective cohort study analyzing a sample of 22 adult patients (aged 18-65) diagnosed with DCM who underwent spinal surgery with IONM in three regional centers in Malaysia between January 2022 and December 2023. 

Inclusion criteria included complete preoperative mJOA scores and the absence of non-degenerative myelopathy causes. Patients’ data were retrospectively extracted from medical records, focusing on baseline MEPs, categorized qualitatively as 'Present' or 'Indeterminate', and preoperative mJOA scores (0-17). 

Statistical analysis was performed using the online platform DataTab (https://numiqo.com/). 

The relationship between these variables was assessed through descriptive statistics and a binary logistic regression, modeling the mJOA score as a linear predictor for the binary outcome of MEP presence. The classification threshold was determined using univariate threshold analysis using the closest-to-(0,1) criterion in the ROC curve.

## Results

A total of 22 patients who met the inclusion and exclusion criteria were included in this study, with 15 patients being male and seven patients being female. Tables 1-3 show the descriptive statistics of the patients. The average age was 59.4 (standard deviation (SD) = 11.55). All patients recorded present MEP in the upper limb (UL) (Table 3). Thirty-six percent of patients recorded indeterminate MEP in the lower limb (LL).

**Table 1:** Descriptive statistics of patients' data in this study mJOA: Modified Japanese Orthopaedic Association

	Sex	Frequency	Mean	Std. Deviation	Minimum	Maximum
Age	F	7	62.27	11.12	45	73
	M	15	58.07	12.23	42	82
mJOA	F	7	10.43	2.64	5	13
	M	15	12.07	2.49	7	15

**Table 2:** Distribution of the mJOA score and MEP of the lower limb mJOA: Modified Japanese Orthopaedic Association; MEP: motor evoked potential

MEP		mJOA score			
Lower Limb	Frequency	Mean	Std. Deviation	Minimum	Maximum
Present	14	12	2.69	5	15
Indistinction	8	10.75	2.38	7	15

**Table 3:** Distribution of the mJOA score and MEP of the upper limb mJOA: Modified Japanese Orthopaedic Association; MEP: motor evoked potential

MEP		mJOA score			
Upper Limb	Frequency	Mean	Std. Deviation	Minimum	Maximum
Present	22	11.55	2.6	5	15

Across MEP UL Present, MEP LL Indeterminate, and MEP LL Present, the average mJOA scores lie roughly between 11.5 and 12.0 with moderate spread (SD ~2.3 to 2.7). The data distributions are fairly similar across groups, each exhibiting a mild negative skew. Variation exists in the LL, with a mean mJOA score of 11.68 (SD = 2.38) for the LL in the indeterminate group and 12.00 (SD = 2.69) in the present group. 

**Figure 1:**
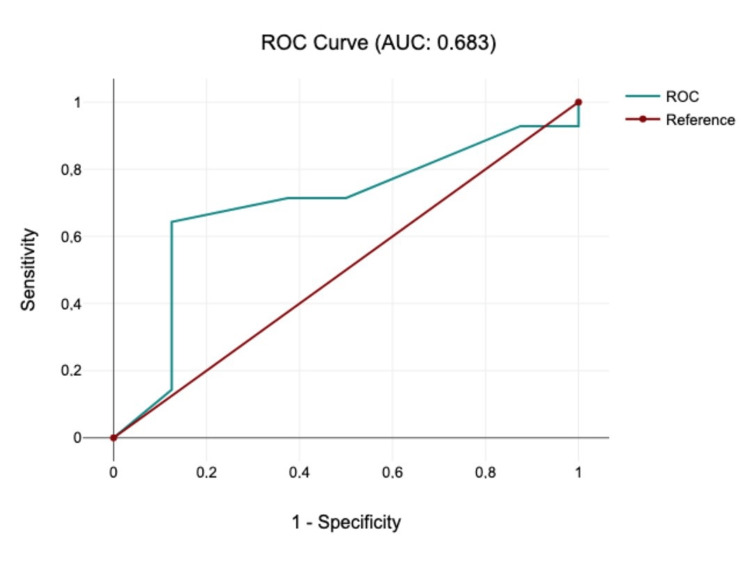
ROC curve (area under the curve = 0.683, sensitivity = 0.643, specificity = 0.875)

Univariate threshold analysis was used to determine the classification threshold of 71% with an mJOA value of 13 (sensitivity=0.643, specificity=0.875). Binary logistic regression analysis (Figure 1) showed an odds ratio of 1.22, indicating that one unit increase in the MJOA score will increase the odds of the dependent variable, Present, by 1.22 times. This suggests a 21.76% increase in the odds of the outcome Present for each additional unit of the mJOA score. However, the p-value of 0.281 is above the conventional 0.05 threshold, suggesting that the mJOA score is not statistically significant at the 5% level. 

## Discussion

In recent years, public healthcare systems have undergone tremendous fiscal pressure. Part of this issue is caused by the increasing use of advanced technology in spine surgery. One such example is the increasing use of IONM in DCM surgery, which has added cost. The exact indication for IONM has undergone scrutiny, given its substantial cost and uncertain impact on neurological outcomes. 

In DCM surgery, the problem is 3 fold: non-linear cost-benefit ratio, inconsistent evidence for reduction in neurologic injury, and lower sensitivity and specificity of IONM in myelopathic cord. Multiple studies have shown that increased use of IONM leads to an increase in total payment cost and does not decrease the rate of neurologic injury [2,3]. Other studies have also shown that the incidence of neurologic injury does not significantly differ even when IONM is not used [4]. 

In the case of myelopathy, IONM and MEP showed reduction in efficacy [5,6]. The sensitivity and specificity of IONM for predicting postoperative neurologic deficit are 42.86% and 68.55%, respectively, in patients undergoing surgery for DCM [7]. IONM is also less predictive of the neurologic outcome and exhibits variability in its baseline signal [3,8]. This is consistent with our finding, which showed that 36% of patients have an indeterminate signal in the LLs. 

The underlying mechanism for reduced accuracy and indeterminate signal could be due to slowed conduction in demyelinated corticospinal fibers, conduction along other oligosynaptic pathways, or reduction of size and synchrony of corticospinal volleys reaching the anterior horn cells [9]. 

The MEP specifically has lower monitorability in patients with severe preoperative myelopathy due to poor baseline MEP signals [10]. Inability to establish a baseline signal will render intraoperative neurophysiologic monitoring ineffective. These cases may represent an inefficient increase in healthcare cost especially when the public fund is already stretched thin. Therefore, identification of a preoperative marker to predict the usefulness of IONM in myelopathic conditions is important. 

The mJOA score is widely used in clinical assessment of DCM with a role in assessing prognosis and guiding treatment [11,12]. Other reports have shown a statistically significant correlation between the mJOA score and neurophysiologic studies as well as clinical features [13,14]. In our study, using the ROC curve, we determined that when the mJOA score is 13, the probability of baseline MEP being present is 71% (Figure 1). We also established that increasing the mJOA score increases the likelihood of the present baseline MEP demonstrating a correlation between the mJOA score and baseline MEP. However, due to our small sample size, the power of our statistical test is low, and the result was not significant. Using a single clinical measurement tool may also include bias in our conclusion, as DCM is a complex disease and involves many confounding factors. To overcome this, future studies may include a multifactorial score system that includes the mJOA score and the Muscle Research Council Scale for Muscle Strength (MRC) to predict the presence of IONM baseline. 

An interesting finding is that the signal in the UL was present in all patients, while the LL signal exhibited variability in our patients. Other studies have noted prolonged motor conduction latency in the UL than in the LL in cervical myelopathy [15]. Neurophysiologic studies also show greater sensitivity in the LL than the UL [16]. In our study, the UL signal may be explained by four reasons: (i) this represents a false positive finding; (ii) due to the small sample of this study, the patients' data may not represent patients with abnormal signals in the UL; (iii) the mJOA score is a compound scoring assessing the severity of cervical myelopathy, while the IONM measures the presence of signal in the UL and the LL; (iv) the possibility that there is a non-linear relationship between the severity of clinical scores, such as mJOA, and neurophysiologic signals. This relationship might be different in the UL and the LL. Due to this, we performed univariate analysis and binary logistic regression analysis on the LL, and not the UL. This may be overcome by using other clinical markers that represent ULs' and LLs' neurologic function separately, such as the MRC scale. A bigger sample size would also provide a wider range of neurologic severity and include those with an indeterminate signal in the ULs. This also represents opportunities to look into the relationship between the severity of neurologic deficits and neurophysiologic signals in different regions.

## Conclusions

A higher preoperative mJOA score correlates with a higher probability of measurable baseline MEP, as evidenced by an odds ratio of 1.22. This demonstrated a correlation between the mJOA score and the presence of baseline MEP in DCM patients. However, due to the small sample size, this finding was not statistically significant. Further study with a greater number of patients will provide stronger statistical power before embarking on prospective validation studies. We also suggest investigating other preoperative clinical parameters, for example, the MRC scale. Combining different clinical scores might provide a more robust model to predict the presence of baseline MEP.

## References

[1] Malhotra NR, Shaffrey CI (2010). Intraoperative electrophysiological monitoring in spine surgery. Spine (Phila Pa 1976).

[2] Clark AJ, Safaee M, Chou D, Weinstein PR, Molinaro AM, Clark JP 3rd, Mummaneni PV (2016). Comparative sensitivity of intraoperative motor evoked potential monitoring in predicting postoperative neurologic deficits: nondegenerative versus degenerative myelopathy. Global Spine J.

[3] DiMaria S, Wilent WB, Nicholson KJ (2022). Patient factors impacting baseline motor evoked potentials (MEPs) in patients undergoing cervical spine surgery for myelopathy or radiculopathy. Clin Spine Surg.

[4] Philipp LR, Leibold A, Mahtabfar A, Montenegro TS, Gonzalez GA, Harrop JS (2021). Achieving value in spine surgery: 10 major cost contributors. Global Spine J.

[5] Rowe DG, Barrett C, Owolo E (2025). The prevalence of intraoperative neuromonitoring in anterior cervical discectomy and fusion: trends, variances, and value appraisal. Clin Spine Surg.

[6] El Choueiri J, Pellicanò F, Caimi E (2025). Intraoperative neuromonitoring in cervical degenerative spine surgery: a meta-analysis of its impact on neurological outcomes. Neurosurg Rev.

[7] Clark AJ, Ziewacz JE, Safaee M (2013). Intraoperative neuromonitoring with MEPs and prediction of postoperative neurological deficits in patients undergoing surgery for cervical and cervicothoracic myelopathy. Neurosurg Focus.

[8] Gamblin AS, Awad AW, Karsy M (2023). Efficacy of intraoperative neuromonitoring during the treatment of cervical myelopathy. Indian J Neurosurg.

[9] Yu Z, Pan W, Chen J, Peng X, Ling Z, Zou X (2022). Application of electrophysiological measures in degenerative cervical myelopathy. Front Cell Dev Biol.

[10] Wang S, Ren Z, Liu J, Zhang J, Tian Y (2020). The prediction of intraoperative cervical cord function changes by different motor evoked potentials phenotypes in cervical myelopathy patients. BMC Neurol.

[11] Benzel EC, Lancon J, Kesterson L, Hadden T (1991). Cervical laminectomy and dentate ligament section for cervical spondylotic myelopathy. J Spinal Disord.

[12] Bednarík J, Kadanka Z, Vohánka S, Stejskal L, Vlach O, Schröder R (1999). The value of somatosensory- and motor-evoked potentials in predicting and monitoring the effect of therapy in spondylotic cervical myelopathy. Prospective randomized study. Spine (Phila Pa 1976).

[13] Rikita T, Tanaka N, Nakanishi K (2017). The relationship between central motor conduction time and spinal cord compression in patients with cervical spondylotic myelopathy. Spinal Cord.

[14] Yu D, Chang MC, Jeon I, Kim SW (2024). Diagnostic and prognostic significance of preoperative evoked potential tests in degenerative cervical myelopathy. Spine J.

[15] Nardone R, Höller Y, Brigo F (2016). The contribution of neurophysiology in the diagnosis and management of cervical spondylotic myelopathy: a review. Spinal Cord.

[16] Dvorak J, Sutter M, Herdmann J (2003). Cervical myelopathy: clinical and neurophysiological evaluation. Eur Spine J.

